# Simple Method of Testing Quinine

**Published:** 1850-05

**Authors:** 


*Ohio Med. and Surg. Journal.*

---

Simple Method of Testing Quinine.—Our worthy friend, Charles Augustus Smith, Pharmaceutist of Cincinnati, has published the following plan for detecting stearic and margaric acids and spermaceti in sulphate of quinine, by means of chloroform. Six grains of the suspected salt are agitated in a test tube with a fluid drachm of chloroform for two minutes; the sulphate of quinine is then dissolved out by dilute sulphuric acid, the solution separated from the chloroform, which is then washed with distilled water, and suffered to evaporate gradually on a piece of paper. The fatty matter, if present to the extent of ten per cent., will be found on the paper, which will itself have a greasy stain upon it.

*Ohio Med. and Surg. Journal.*

